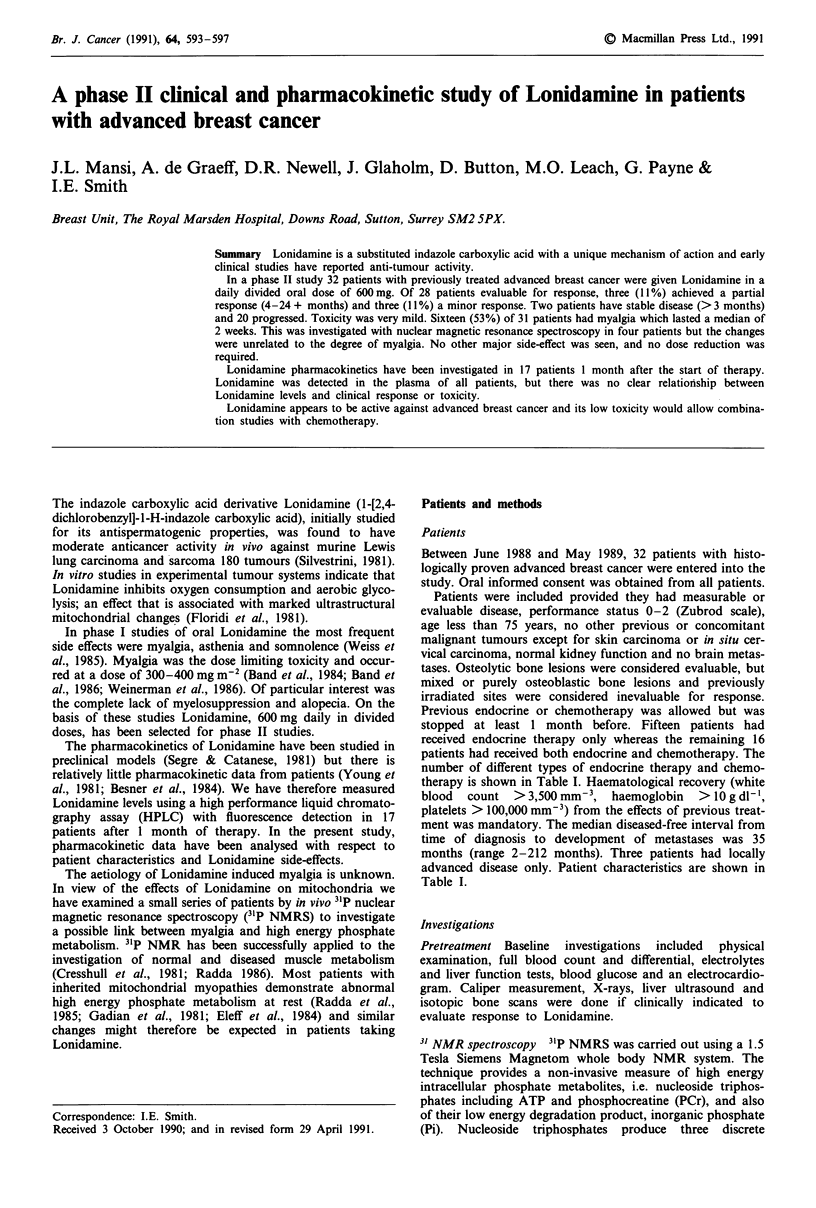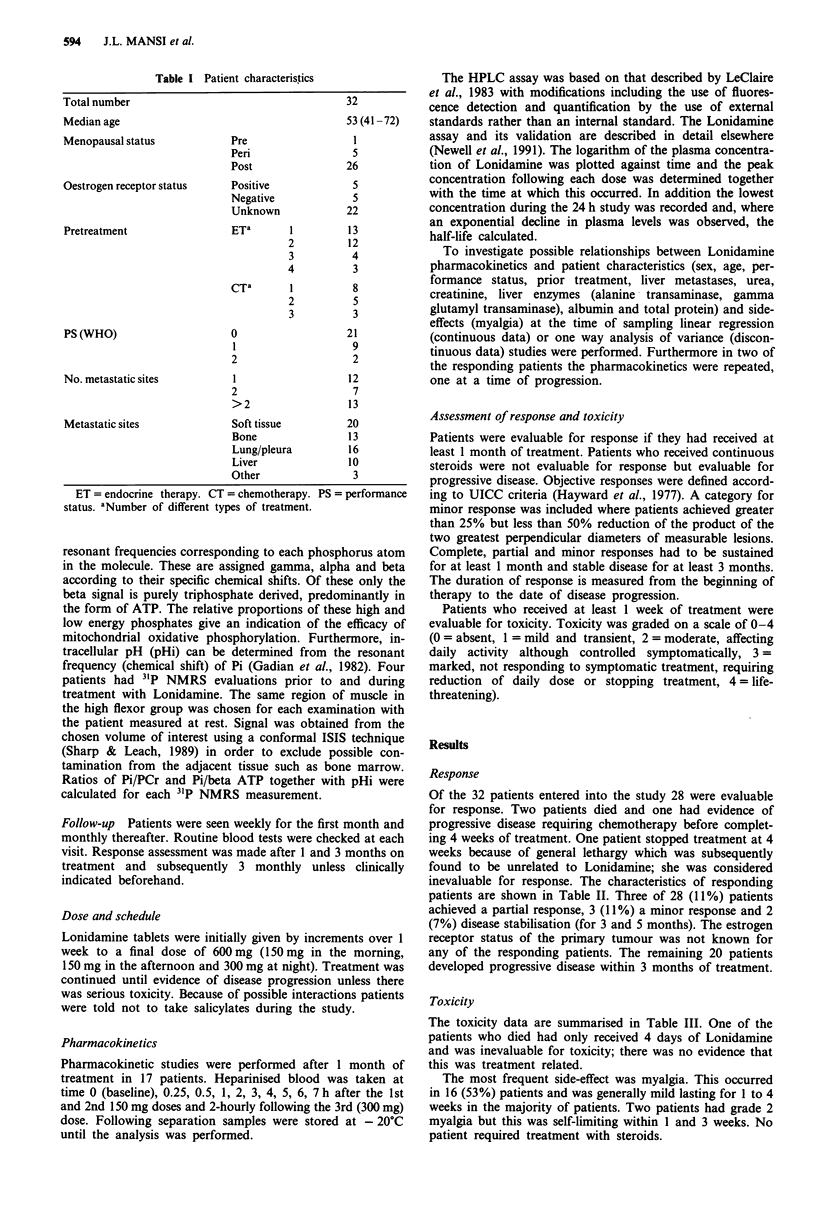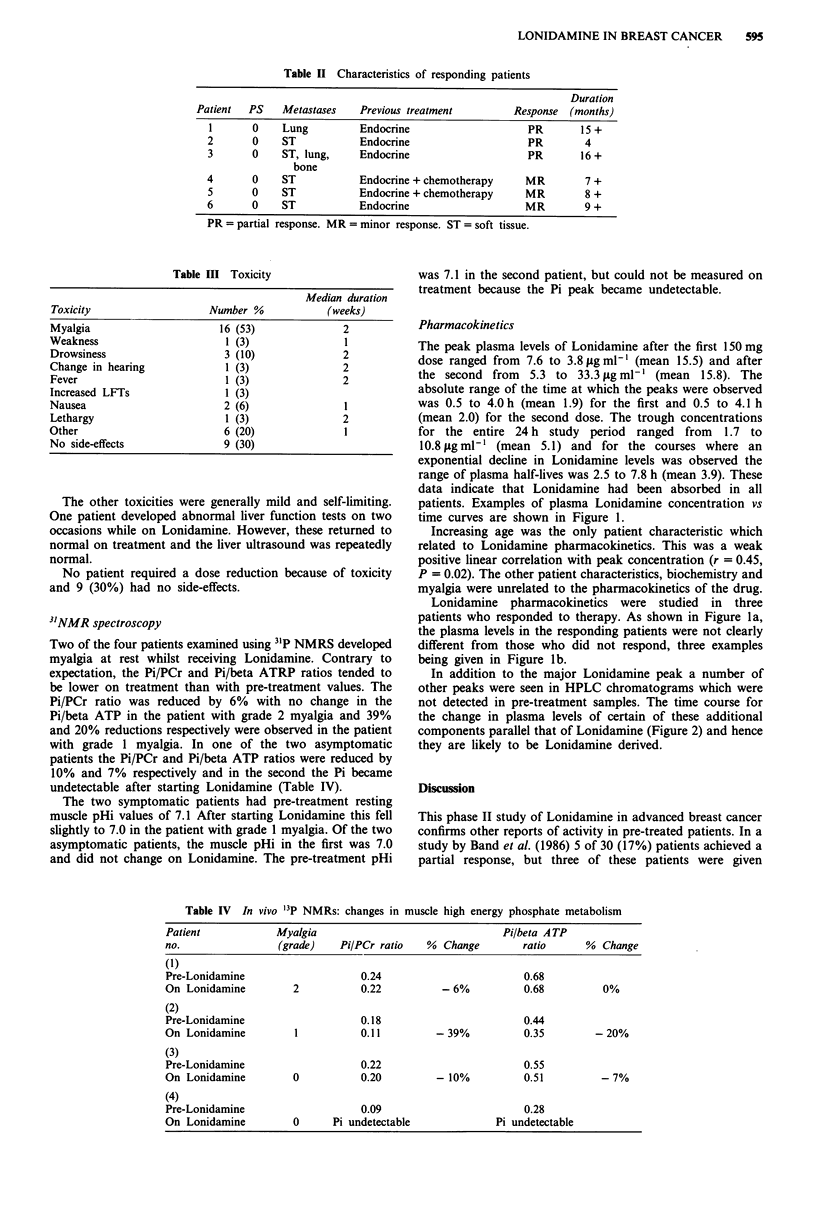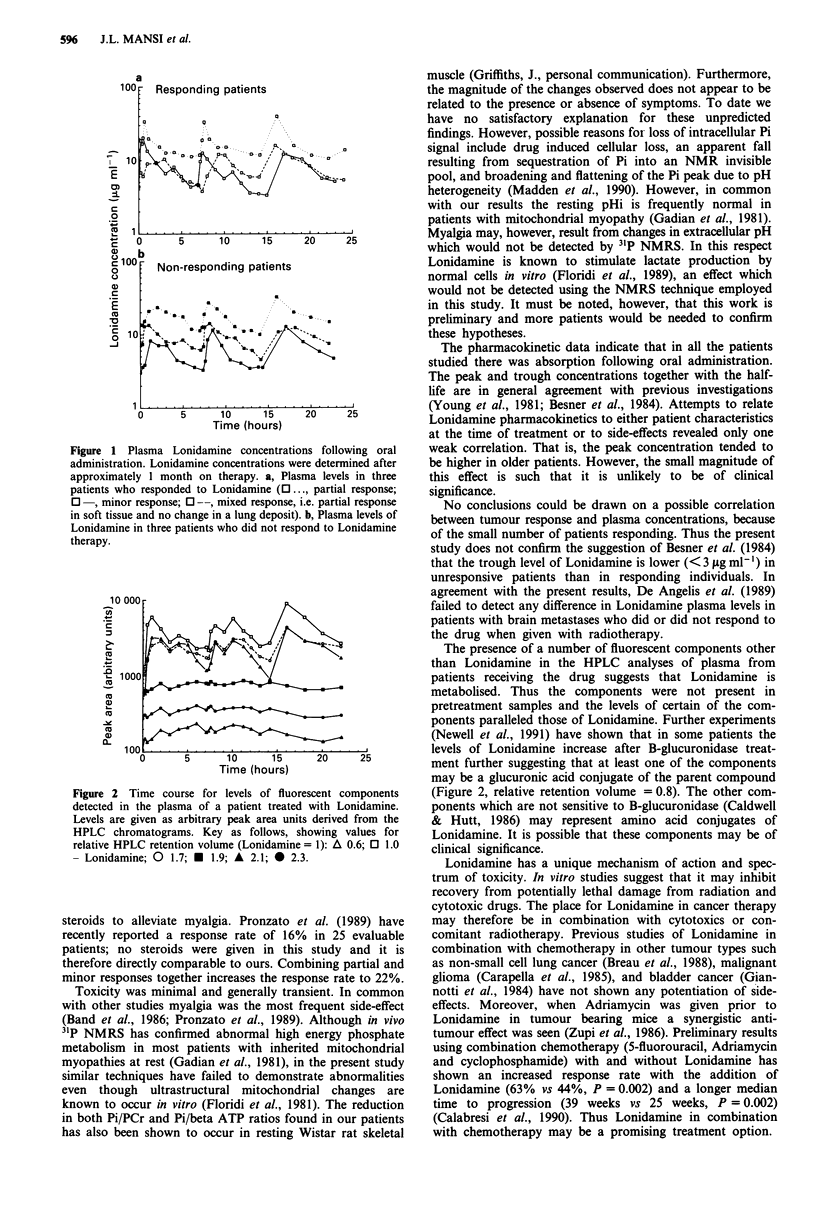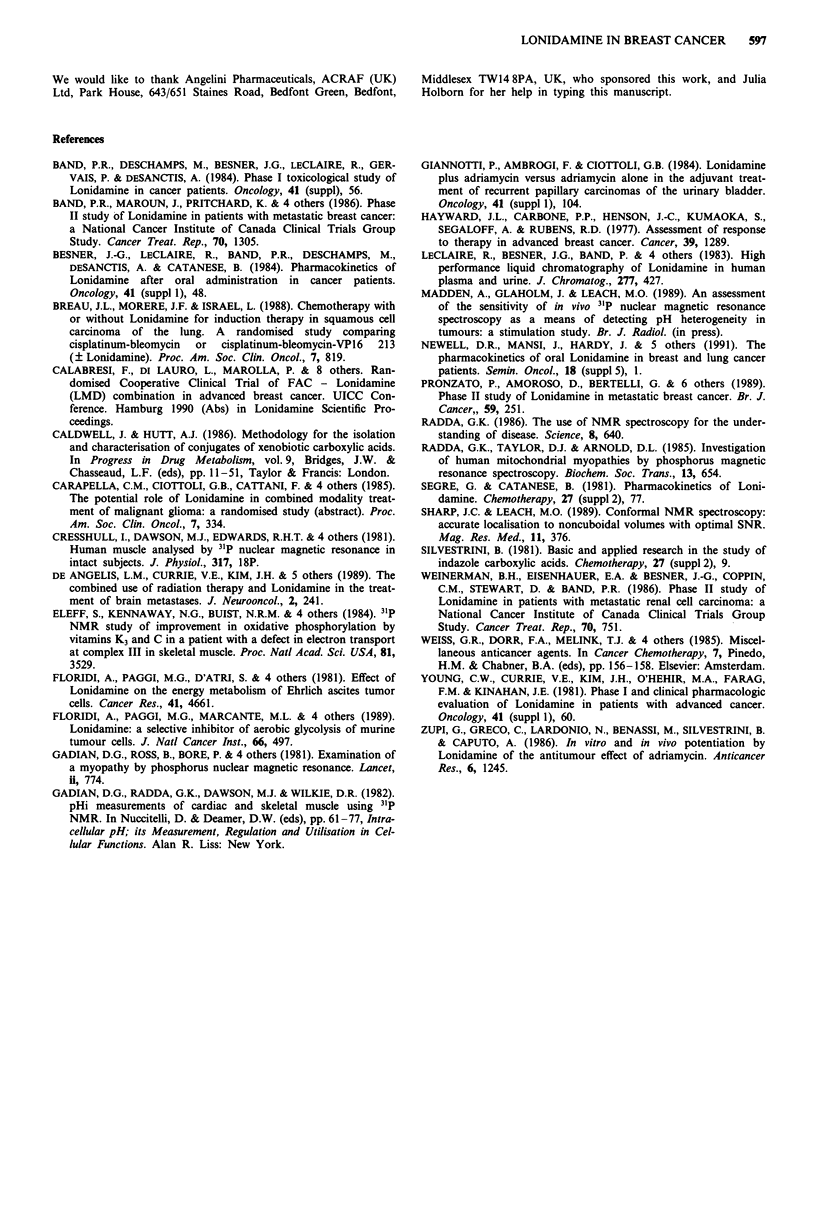# A phase II clinical and pharmacokinetic study of Lonidamine in patients with advanced breast cancer.

**DOI:** 10.1038/bjc.1991.356

**Published:** 1991-09

**Authors:** J. L. Mansi, A. de Graeff, D. R. Newell, J. Glaholm, D. Button, M. O. Leach, G. Payne, I. E. Smith

**Affiliations:** Breast Unit, Royal Marsden Hospital, Sutton, Surrey.

## Abstract

Lonidamine is a substituted indazole carboxylic acid with a unique mechanism of action and early clinical studies have reported anti-tumour activity. In a phase II study 32 patients with previously treated advanced breast cancer were given Lonidamine in a daily divided oral dose of 600 mg. Of 28 patients evaluable for response, three (11%) achieved a partial response (4-24+ months) and three (11%) a minor response. Two patients have stable disease (greater than 3 months) and 20 progressed. Toxicity was very mild. Sixteen (53%) of 31 patients had myalgia which lasted a median of 2 weeks. This was investigated with nuclear magnetic resonance spectroscopy in four patients but the changes were unrelated to the degree of myalgia. No other major side-effect was seen, and no dose reduction was required. Lonidamine pharmacokinetics have been investigated in 17 patients 1 month after the start of therapy. Lonidamine was detected in the plasma of all patients, but there was no clear relationship between Lonidamine levels and clinical response or toxicity. Lonidamine appears to be active against advanced breast cancer and its low toxicity would allow combination studies with chemotherapy.


Br. J. Cancer (1991), 64, 593-597 © Macmillan Press Ltd., 1991

### A phase II clinical and pharmacokinetic study of Lonidamine in patients with advanced breast cancer

J.L. Mansi, A. de Graeff, D.R. Newell, J. Glaholm, D. Button, M.O. Leach, G. Payne & I.E. Smith

*Breast Unit, The Royal Marsden Hospital, Downs Road, Sutton, Surrey SM2 5PX.*

**Summary** Lonidamine is a substituted indazole carboxylic acid with a unique mechanism of action and early clinical studies have reported anti-tumour activity.

In a phase II study 32 patients with previously treated advanced breast cancer were given Lonidamine in a daily divided oral dose of 600 mg. Of 28 patients evaluable for response, three (11%) achieved a partial response (4-24 + months) and three (11%) a minor response. Two patients have stable disease (>3 months) and 20 progressed. Toxicity was very mild. Sixteen (53%) of 31 patients had myalgia which lasted a median of 2 weeks. This was investigated with nuclear magnetic resonance spectroscopy in four patients but the changes were unrelated to the degree of myalgia. No other major side-effect was seen, and no dose reduction was required.

Lonidamine pharmacokinetics have been investigated in 17 patients 1 month after the start of therapy. Lonidamine was detected in the plasma of all patients, but there was no clear relationship between Lonidamine levels and clinical response or toxicity.

Lonidamine appears to be active against advanced breast cancer and its low toxicity would allow combination studies with chemotherapy.

The indazole carboxylic acid derivative Lonidamine (1-[2,4-dichlorobenzyl]-1-H-indazole carboxylic acid), initially studied for its antispermatogenic properties, was found to have moderate anticancer activity *in vivo* against murine Lewis lung carcinoma and sarcoma 180 tumours (Silvestrini, 1981). *In vitro* studies in experimental tumour systems indicate that Lonidamine inhibits oxygen consumption and aerobic glycolysis; an effect that is associated with marked ultrastructural mitochondrial changes (Floridi *et al.*, 1981).

In phase I studies of oral Lonidamine the most frequent side effects were myalgia, asthenia and somnolence (Weiss *et al.*, 1985). Myalgia was the dose limiting toxicity and occurred at a dose of 300-400 mg $m^{-2}$ (Band *et al.*, 1984; Band *et al.*, 1986; Weinerman *et al.*, 1986). Of particular interest was the complete lack of myelosuppression and alopecia. On the basis of these studies Lonidamine, 600 mg daily in divided doses, has been selected for phase II studies.

The pharmacokinetics of Lonidamine have been studied in preclinical models (Segre & Catanese, 1981) but there is relatively little pharmacokinetic data from patients (Young *et al.*, 1981; Besner *et al.*, 1984). We have therefore measured Lonidamine levels using a high performance liquid chromatography assay (HPLC) with fluorescence detection in 17 patients after 1 month of therapy. In the present study, pharmacokinetic data have been analysed with respect to patient characteristics and Lonidamine side-effects.

The aetiology of Lonidamine induced myalgia is unknown. In view of the effects of Lonidamine on mitochondria we have examined a small series of patients by *in vivo* $^{31}$P nuclear magnetic resonance spectroscopy ($^{31}$P NMRS) to investigate a possible link between myalgia and high energy phosphate metabolism. $^{31}$P NMR has been successfully applied to the investigation of normal and diseased muscle metabolism (Cresshull *et al.*, 1981; Radda 1986). Most patients with inherited mitochondrial myopathies demonstrate abnormal high energy phosphate metabolism at rest (Radda *et al.*, 1985; Gadian *et al.*, 1981; Eleff *et al.*, 1984) and similar changes might therefore be expected in patients taking Lonidamine.

Correspondence: I.E. Smith.
Received 3 October 1990; and in revised form 29 April 1991.

### Patients and methods

### Patients

Between June 1988 and May 1989, 32 patients with histologically proven advanced breast cancer were entered into the study. Oral informed consent was obtained from all patients.

Patients were included provided they had measurable or evaluable disease, performance status 0-2 (Zubrod scale), age less than 75 years, no other previous or concomitant malignant tumours except for skin carcinoma or *in situ* cervical carcinoma, normal kidney function and no brain metastases. Osteolytic bone lesions were considered evaluable, but mixed or purely osteoblastic bone lesions and previously irradiated sites were considered inevaluable for response. Previous endocrine or chemotherapy was allowed but was stopped at least 1 month before. Fifteen patients had received endocrine therapy only whereas the remaining 16 patients had received both endocrine and chemotherapy. The number of different types of endocrine therapy and chemotherapy is shown in Table I. Haematological recovery (white blood count >3,500 $mm^{-3}$, haemoglobin >10 g $dl^{-1}$, platelets >100,000 $mm^{-3}$) from the effects of previous treatment was mandatory. The median diseased-free interval from time of diagnosis to development of metastases was 35 months (range 2-212 months). Three patients had locally advanced disease only. Patient characteristics are shown in Table I.

### Investigations

*Pretreatment* Baseline investigations included physical examination, full blood count and differential, electrolytes and liver function tests, blood glucose and an electrocardiogram. Caliper measurement, X-rays, liver ultrasound and isotopic bone scans were done if clinically indicated to evaluate response to Lonidamine.

*$^{31}$ NMR spectroscopy* $^{31}$P NMRS was carried out using a 1.5 Tesla Siemens Magnetom whole body NMR system. The technique provides a non-invasive measure of high energy intracellular phosphate metabolites, i.e. nucleoside triphosphates including ATP and phosphocreatine (PCr), and also of their low energy degradation product, inorganic phosphate (Pi). Nucleoside triphosphates produce three discrete

Table I Patient characteristics

| | | | |
|---|---|---|---|
| Total number | | | 32 |
| Median age | | | 53 (41-72) |
| Menopausal status | Pre | | 1 |
| | Peri | | 5 |
| | Post | | 26 |
| Oestrogen receptor status | Positive | | 5 |
| | Negative | | 5 |
| | Unknown | | 22 |
| Pretreatment | ET[a] | 1 | 13 |
| | | 2 | 12 |
| | | 3 | 4 |
| | | 4 | 3 |
| | CT[a] | 1 | 8 |
| | | 2 | 5 |
| | | 3 | 3 |
| PS (WHO) | 0 | | 21 |
| | 1 | | 9 |
| | 2 | | 2 |
| No. metastatic sites | 1 | | 12 |
| | 2 | | 7 |
| | >2 | | 13 |
| Metastatic sites | Soft tissue | | 20 |
| | Bone | | 13 |
| | Lung/pleura | | 16 |
| | Liver | | 10 |
| | Other | | 3 |

ET = endocrine therapy. CT = chemotherapy. PS = performance status. [a]Number of different types of treatment.

resonant frequencies corresponding to each phosphorus atom in the molecule. These are assigned gamma, alpha and beta according to their specific chemical shifts. Of these only the beta signal is purely triphosphate derived, predominantly in the form of ATP. The relative proportions of these high and low energy phosphates give an indication of the efficacy of mitochondrial oxidative phosphorylation. Furthermore, intracellular pH (pHi) can be determined from the resonant frequency (chemical shift) of Pi (Gadian *et al.*, 1982). Four patients had $^{31}$P NMRS evaluations prior to and during treatment with Lonidamine. The same region of muscle in the high flexor group was chosen for each examination with the patient measured at rest. Signal was obtained from the chosen volume of interest using a conformal ISIS technique (Sharp & Leach, 1989) in order to exclude possible contamination from the adjacent tissue such as bone marrow. Ratios of Pi/PCr and Pi/beta ATP together with pHi were calculated for each $^{31}$P NMRS measurement.

*Follow-up* Patients were seen weekly for the first month and monthly thereafter. Routine blood tests were checked at each visit. Response assessment was made after 1 and 3 months on treatment and subsequently 3 monthly unless clinically indicated beforehand.

*Dose and schedule*

Lonidamine tablets were initially given by increments over 1 week to a final dose of 600 mg (150 mg in the morning, 150 mg in the afternoon and 300 mg at night). Treatment was continued until evidence of disease progression unless there was serious toxicity. Because of possible interactions patients were told not to take salicylates during the study.

*Pharmacokinetics*

Pharmacokinetic studies were performed after 1 month of treatment in 17 patients. Heparinised blood was taken at time 0 (baseline), 0.25, 0.5, 1, 2, 3, 4, 5, 6, 7 h after the 1st and 2nd 150 mg doses and 2-hourly following the 3rd (300 mg) dose. Following separation samples were stored at − 20°C until the analysis was performed.

The HPLC assay was based on that described by LeClaire *et al.*, 1983 with modifications including the use of fluorescence detection and quantification by the use of external standards rather than an internal standard. The Lonidamine assay and its validation are described in detail elsewhere (Newell *et al.*, 1991). The logarithm of the plasma concentration of Lonidamine was plotted against time and the peak concentration following each dose was determined together with the time at which this occurred. In addition the lowest concentration during the 24 h study was recorded and, where an exponential decline in plasma levels was observed, the half-life calculated.

To investigate possible relationships between Lonidamine pharmacokinetics and patient characteristics (sex, age, performance status, prior treatment, liver metastases, urea, creatinine, liver enzymes (alanine transaminase, gamma glutamyl transaminase), albumin and total protein) and side-effects (myalgia) at the time of sampling linear regression (continuous data) or one way analysis of variance (discontinuous data) studies were performed. Furthermore in two of the responding patients the pharmacokinetics were repeated, one at a time of progression.

*Assessment of response and toxicity*

Patients were evaluable for response if they had received at least 1 month of treatment. Patients who received continuous steroids were not evaluable for response but evaluable for progressive disease. Objective responses were defined according to UICC criteria (Hayward *et al.*, 1977). A category for minor response was included where patients achieved greater than 25% but less than 50% reduction of the product of the two greatest perpendicular diameters of measurable lesions. Complete, partial and minor responses had to be sustained for at least 1 month and stable disease for at least 3 months. The duration of response is measured from the beginning of therapy to the date of disease progression.

Patients who received at least 1 week of treatment were evaluable for toxicity. Toxicity was graded on a scale of 0–4 (0 = absent, 1 = mild and transient, 2 = moderate, affecting daily activity although controlled symptomatically, 3 = marked, not responding to symptomatic treatment, requiring reduction of daily dose or stopping treatment, 4 = life-threatening).

### Results

*Response*

Of the 32 patients entered into the study 28 were evaluable for response. Two patients died and one had evidence of progressive disease requiring chemotherapy before completing 4 weeks of treatment. One patient stopped treatment at 4 weeks because of general lethargy which was subsequently found to be unrelated to Lonidamine; she was considered inevaluable for response. The characteristics of responding patients are shown in Table II. Three of 28 (11%) patients achieved a partial response, 3 (11%) a minor response and 2 (7%) disease stabilisation (for 3 and 5 months). The estrogen receptor status of the primary tumour was not known for any of the responding patients. The remaining 20 patients developed progressive disease within 3 months of treatment.

*Toxicity*

The toxicity data are summarised in Table III. One of the patients who died had only received 4 days of Lonidamine and was inevaluable for toxicity; there was no evidence that this was treatment related.

The most frequent side-effect was myalgia. This occurred in 16 (53%) patients and was generally mild lasting for 1 to 4 weeks in the majority of patients. Two patients had grade 2 myalgia but this was self-limiting within 1 and 3 weeks. No patient required treatment with steroids.

**Table II** Characteristics of responding patients

| Patient | PS | Metastases | Previous treatment | Response | Duration (months) |
|---|---|---|---|---|---|
| 1 | 0 | Lung | Endocrine | PR | 15+ |
| 2 | 0 | ST | Endocrine | PR | 4 |
| 3 | 0 | ST, lung, bone | Endocrine | PR | 16+ |
| 4 | 0 | ST | Endocrine + chemotherapy | MR | 7+ |
| 5 | 0 | ST | Endocrine + chemotherapy | MR | 8+ |
| 6 | 0 | ST | Endocrine | MR | 9+ |

PR = partial response. MR = minor response. ST = soft tissue.

**Table III** Toxicity

| Toxicity | Number % | Median duration (weeks) |
|---|---|---|
| Myalgia | 16 (53) | 2 |
| Weakness | 1 (3) | 1 |
| Drowsiness | 3 (10) | 2 |
| Change in hearing | 1 (3) | 2 |
| Fever | 1 (3) | 2 |
| Increased LFTs | 1 (3) | |
| Nausea | 2 (6) | 1 |
| Lethargy | 1 (3) | 2 |
| Other | 6 (20) | 1 |
| No side-effects | 9 (30) | |

The other toxicities were generally mild and self-limiting. One patient developed abnormal liver function tests on two occasions while on Lonidamine. However, these returned to normal on treatment and the liver ultrasound was repeatedly normal.

No patient required a dose reduction because of toxicity and 9 (30%) had no side-effects.

*$^{31}$NMR spectroscopy*

Two of the four patients examined using $^{31}$P NMRS developed myalgia at rest whilst receiving Lonidamine. Contrary to expectation, the Pi/PCr and Pi/beta ATRP ratios tended to be lower on treatment than with pre-treatment values. The Pi/PCr ratio was reduced by 6% with no change in the Pi/beta ATP in the patient with grade 2 myalgia and 39% and 20% reductions respectively were observed in the patient with grade 1 myalgia. In one of the two asymptomatic patients the Pi/PCr and Pi/beta ATP ratios were reduced by 10% and 7% respectively and in the second the Pi became undetectable after starting Lonidamine (Table IV).

The two symptomatic patients had pre-treatment resting muscle pHi values of 7.1 After starting Lonidamine this fell slightly to 7.0 in the patient with grade 1 myalgia. Of the two asymptomatic patients, the muscle pHi in the first was 7.0 and did not change on Lonidamine. The pre-treatment pHi was 7.1 in the second patient, but could not be measured on treatment because the Pi peak became undetectable.

*Pharmacokinetics*

The peak plasma levels of Lonidamine after the first 150 mg dose ranged from 7.6 to 3.8 $\mu g\,ml^{-1}$ (mean 15.5) and after the second from 5.3 to 33.3 $\mu g\,ml^{-1}$ (mean 15.8). The absolute range of the time at which the peaks were observed was 0.5 to 4.0 h (mean 1.9) for the first and 0.5 to 4.1 h (mean 2.0) for the second dose. The trough concentrations for the entire 24 h study period ranged from 1.7 to 10.8 $\mu g\,ml^{-1}$ (mean 5.1) and for the courses where an exponential decline in Lonidamine levels was observed the range of plasma half-lives was 2.5 to 7.8 h (mean 3.9). These data indicate that Lonidamine had been absorbed in all patients. Examples of plasma Lonidamine concentration *vs* time curves are shown in Figure 1.

Increasing age was the only patient characteristic which related to Lonidamine pharmacokinetics. This was a weak positive linear correlation with peak concentration ($r = 0.45$, $P = 0.02$). The other patient characteristics, biochemistry and myalgia were unrelated to the pharmacokinetics of the drug.

Lonidamine pharmacokinetics were studied in three patients who responded to therapy. As shown in Figure 1a, the plasma levels in the responding patients were not clearly different from those who did not respond, three examples being given in Figure 1b.

In addition to the major Lonidamine peak a number of other peaks were seen in HPLC chromatograms which were not detected in pre-treatment samples. The time course for the change in plasma levels of certain of these additional components parallel that of Lonidamine (Figure 2) and hence they are likely to be Lonidamine derived.

### Discussion

This phase II study of Lonidamine in advanced breast cancer confirms other reports of activity in pre-treated patients. In a study by Band *et al.* (1986) 5 of 30 (17%) patients achieved a partial response, but three of these patients were given

**Table IV** *In vivo* $^{13}$P NMRs: changes in muscle high energy phosphate metabolism

| Patient no. | Myalgia (grade) | Pi/PCr ratio | % Change | Pi/beta ATP ratio | % Change |
|---|---|---|---|---|---|
| (1) | | | | | |
| Pre-Lonidamine | | 0.24 | | 0.68 | |
| On Lonidamine | 2 | 0.22 | − 6% | 0.68 | 0% |
| (2) | | | | | |
| Pre-Lonidamine | | 0.18 | | 0.44 | |
| On Lonidamine | 1 | 0.11 | − 39% | 0.35 | − 20% |
| (3) | | | | | |
| Pre-Lonidamine | | 0.22 | | 0.55 | |
| On Lonidamine | 0 | 0.20 | − 10% | 0.51 | − 7% |
| (4) | | | | | |
| Pre-Lonidamine | | 0.09 | | 0.28 | |
| On Lonidamine | 0 | Pi undetectable | | Pi undetectable | |

**Figure 1** Plasma Lonidamine concentrations following oral administration. Lonidamine concentrations were determined after approximately 1 month on therapy. **a**, Plasma levels in three patients who responded to Lonidamine (□..., partial response; □—, minor response; □--, mixed response, i.e. partial response in soft tissue and no change in a lung deposit). **b**, Plasma levels of Lonidamine in three patients who did not respond to Lonidamine therapy.

**Figure 2** Time course for levels of fluorescent components detected in the plasma of a patient treated with Lonidamine. Levels are given as arbitrary peak area units derived from the HPLC chromatograms. Key as follows, showing values for relative HPLC retention volume (Lonidamine = 1): △ 0.6; □ 1.0 – Lonidamine; ○ 1.7; ■ 1.9; ▲ 2.1; ● 2.3.

steroids to alleviate myalgia. Pronzato *et al.* (1989) have recently reported a response rate of 16% in 25 evaluable patients; no steroids were given in this study and it is therefore directly comparable to ours. Combining partial and minor responses together increases the response rate to 22%.

Toxicity was minimal and generally transient. In common with other studies myalgia was the most frequent side-effect (Band *et al.*, 1986; Pronzato *et al.*, 1989). Although *in vivo* $^{31}P$ NMRS has confirmed abnormal high energy phosphate metabolism in most patients with inherited mitochondrial myopathies at rest (Gadian *et al.*, 1981), in the present study similar techniques have failed to demonstrate abnormalities even though ultrastructural mitochondrial changes are known to occur *in vitro* (Floridi *et al.*, 1981). The reduction in both Pi/PCr and Pi/beta ATP ratios found in our patients has also been shown to occur in resting Wistar rat skeletal muscle (Griffiths, J., personal communication). Furthermore, the magnitude of the changes observed does not appear to be related to the presence or absence of symptoms. To date we have no satisfactory explanation for these unpredicted findings. However, possible reasons for loss of intracellular Pi signal include drug induced cellular loss, an apparent fall resulting from sequestration of Pi into an NMR invisible pool, and broadening and flattening of the Pi peak due to pH heterogeneity (Madden *et al.*, 1990). However, in common with our results the resting pHi is frequently normal in patients with mitochondrial myopathy (Gadian *et al.*, 1981). Myalgia may, however, result from changes in extracellular pH which would not be detected by $^{31}P$ NMRS. In this respect Lonidamine is known to stimulate lactate production by normal cells *in vitro* (Floridi *et al.*, 1989), an effect which would not be detected using the NMRS technique employed in this study. It must be noted, however, that this work is preliminary and more patients would be needed to confirm these hypotheses.

The pharmacokinetic data indicate that in all the patients studied there was absorption following oral administration. The peak and trough concentrations together with the half-life are in general agreement with previous investigations (Young *et al.*, 1981; Besner *et al.*, 1984). Attempts to relate Lonidamine pharmacokinetics to either patient characteristics at the time of treatment or to side-effects revealed only one weak correlation. That is, the peak concentration tended to be higher in older patients. However, the small magnitude of this effect is such that it is unlikely to be of clinical significance.

No conclusions could be drawn on a possible correlation between tumour response and plasma concentrations, because of the small number of patients responding. Thus the present study does not confirm the suggestion of Besner *et al.* (1984) that the trough level of Lonidamine is lower ($<3\ \mu g\ ml^{-1}$) in unresponsive patients than in responding individuals. In agreement with the present results, De Angelis *et al.* (1989) failed to detect any difference in Lonidamine plasma levels in patients with brain metastases who did or did not respond to the drug when given with radiotherapy.

The presence of a number of fluorescent components other than Lonidamine in the HPLC analyses of plasma from patients receiving the drug suggests that Lonidamine is metabolised. Thus the components were not present in pretreatment samples and the levels of certain of the components paralleled those of Lonidamine. Further experiments (Newell *et al.*, 1991) have shown that in some patients the levels of Lonidamine increase after B-glucuronidase treatment further suggesting that at least one of the components may be a glucuronic acid conjugate of the parent compound (Figure 2, relative retention volume = 0.8). The other components which are not sensitive to B-glucuronidase (Caldwell & Hutt, 1986) may represent amino acid conjugates of Lonidamine. It is possible that these components may be of clinical significance.

Lonidamine has a unique mechanism of action and spectrum of toxicity. *In vitro* studies suggest that it may inhibit recovery from potentially lethal damage from radiation and cytotoxic drugs. The place for Lonidamine in cancer therapy may therefore be in combination with cytotoxics or concomitant radiotherapy. Previous studies of Lonidamine in combination with chemotherapy in other tumour types such as non-small cell lung cancer (Breau *et al.*, 1988), malignant glioma (Carapella *et al.*, 1985), and bladder cancer (Giannotti *et al.*, 1984) have not shown any potentiation of side-effects. Moreover, when Adriamycin was given prior to Lonidamine in tumour bearing mice a synergistic anti-tumour effect was seen (Zupi *et al.*, 1986). Preliminary results using combination chemotherapy (5-fluorouracil, Adriamycin and cyclophosphamide) with and without Lonidamine has shown an increased response rate with the addition of Lonidamine (63% *vs* 44%, $P = 0.002$) and a longer median time to progression (39 weeks *vs* 25 weeks, $P = 0.002$) (Calabresi *et al.*, 1990). Thus Lonidamine in combination with chemotherapy may be a promising treatment option.

We would like to thank Angelini Pharmaceuticals, ACRAF (UK) Ltd, Park House, 643/651 Staines Road, Bedfont Green, Bedfont, Middlesex TW14 8PA, UK, who sponsored this work, and Julia Holborn for her help in typing this manuscript.

### References

BAND, P.R., DESCHAMPS, M., BESNER, J.G., LECLAIRE, R., GERVAIS, P. & DESANCTIS, A. (1984). Phase I toxicological study of Lonidamine in cancer patients. *Oncology*, **41** (suppl), 56.

BAND, P.R., MAROUN, J., PRITCHARD, K. & 4 others (1986). Phase II study of Lonidamine in patients with metastatic breast cancer: a National Cancer Institute of Canada Clinical Trials Group Study. *Cancer Treat. Rep.*, **70**, 1305.

BESNER, J.-G., LECLAIRE, R., BAND, P.R., DESCHAMPS, M., DESANCTIS, A. & CATANESE, B. (1984). Pharmacokinetics of Lonidamine after oral administration in cancer patients. *Oncology*, **41** (suppl 1), 48.

BREAU, J.L., MORERE, J.F. & ISRAEL, L. (1988). Chemotherapy with or without Lonidamine for induction therapy in squamous cell carcinoma of the lung. A randomised study comparing cisplatinum-bleomycin or cisplatinum-bleomycin-VP16 213 (± Lonidamine). *Proc. Am. Soc. Clin. Oncol.*, **7**, 819.

CALABRESI, F., DI LAURO, L., MAROLLA, P. & 8 others. Randomised Cooperative Clinical Trial of FAC – Lonidamine (LMD) combination in advanced breast cancer. UICC Conference. Hamburg 1990 (Abs) in Lonidamine Scientific Proceedings.

CALDWELL, J. & HUTT, A.J. (1986). Methodology for the isolation and characterisation of conjugates of xenobiotic carboxylic acids. In *Progress in Drug Metabolism*, vol. 9, Bridges, J.W. & Chasseaud, L.F. (eds), pp. 11–51, Taylor & Francis: London.

CARAPELLA, C.M., CIOTTOLI, G.B., CATTANI, F. & 4 others (1985). The potential role of Lonidamine in combined modality treatment of malignant glioma: a randomised study (abstract). *Proc. Am. Soc. Clin. Oncol.*, **7**, 334.

CRESSHULL, I., DAWSON, M.J., EDWARDS, R.H.T. & 4 others (1981). Human muscle analysed by $^{31}P$ nuclear magnetic resonance in intact subjects. *J. Physiol.*, **317**, 18P.

DE ANGELIS, L.M., CURRIE, V.E., KIM, J.H. & 5 others (1989). The combined use of radiation therapy and Lonidamine in the treatment of brain metastases. *J. Neurooncol.*, **2**, 241.

ELEFF, S., KENNAWAY, N.G., BUIST, N.R.M. & 4 others (1984). $^{31}P$ NMR study of improvement in oxidative phosphorylation by vitamins $K_3$ and C in a patient with a defect in electron transport at complex III in skeletal muscle. *Proc. Natl Acad. Sci. USA*, **81**, 3529.

FLORIDI, A., PAGGI, M.G., D'ATRI, S. & 4 others (1981). Effect of Lonidamine on the energy metabolism of Ehrlich ascites tumor cells. *Cancer Res.*, **41**, 4661.

FLORIDI, A., PAGGI, M.G., MARCANTE, M.L. & 4 others (1989). Lonidamine: a selective inhibitor of aerobic glycolysis of murine tumour cells. *J. Natl Cancer Inst.*, **66**, 497.

GADIAN, D.G., ROSS, B., BORE, P. & 4 others (1981). Examination of a myopathy by phosphorus nuclear magnetic resonance. *Lancet*, **ii**, 774.

GADIAN, D.G., RADDA, G.K., DAWSON, M.J. & WILKIE, D.R. (1982). pHi measurements of cardiac and skeletal muscle using $^{31}P$ NMR. In Nuccitelli, D. & Deamer, D.W. (eds), pp. 61–77, *Intracellular pH; its Measurement, Regulation and Utilisation in Cellular Functions*. Alan R. Liss: New York.

GIANNOTTI, P., AMBROGI, F. & CIOTTOLI, G.B. (1984). Lonidamine plus adriamycin versus adriamycin alone in the adjuvant treatment of recurrent papillary carcinomas of the urinary bladder. *Oncology*, **41** (suppl 1), 104.

HAYWARD, J.L., CARBONE, P.P., HENSON, J.-C., KUMAOKA, S., SEGALOFF, A. & RUBENS, R.D. (1977). Assessment of response to therapy in advanced breast cancer. *Cancer*, **39**, 1289.

LECLAIRE, R., BESNER, J.G., BAND, P. & 4 others (1983). High performance liquid chromatography of Lonidamine in human plasma and urine. *J. Chromatog.*, **277**, 427.

MADDEN, A., GLAHOLM, J. & LEACH, M.O. (1989). An assessment of the sensitivity of *in vivo* $^{31}P$ nuclear magnetic resonance spectroscopy as a means of detecting pH heterogeneity in tumours: a stimulation study. *Br. J. Radiol.* (in press).

NEWELL, D.R., MANSI, J., HARDY, J. & 5 others (1991). The pharmacokinetics of oral Lonidamine in breast and lung cancer patients. *Semin. Oncol.*, **18** (suppl 5), 1.

PRONZATO, P., AMOROSO, D., BERTELLI, G. & 6 others (1989). Phase II study of Lonidamine in metastatic breast cancer. *Br. J. Cancer*,, **59**, 251.

RADDA, G.K. (1986). The use of NMR spectroscopy for the understanding of disease. *Science*, **8**, 640.

RADDA, G.K., TAYLOR, D.J. & ARNOLD, D.L. (1985). Investigation of human mitochondrial myopathies by phosphorus magnetic resonance spectroscopy. *Biochem. Soc. Trans.*, **13**, 654.

SEGRE, G. & CATANESE, B. (1981). Pharmacokinetics of Lonidamine. *Chemotherapy*, **27** (suppl 2), 77.

SHARP, J.C. & LEACH, M.O. (1989). Conformal NMR spectroscopy: accurate localisation to noncuboidal volumes with optimal SNR. *Mag. Res. Med.*, **11**, 376.

SILVESTRINI, B. (1981). Basic and applied research in the study of indazole carboxylic acids. *Chemotherapy*, **27** (suppl 2), 9.

WEINERMAN, B.H., EISENHAUER, E.A. & BESNER, J.-G., COPPIN, C.M., STEWART, D. & BAND, P.R. (1986). Phase II study of Lonidamine in patients with metastatic renal cell carcinoma: a National Cancer Institute of Canada Clinical Trials Group Study. *Cancer Treat. Rep.*, **70**, 751.

WEISS, G.R., DORR, F.A., MELINK, T.J. & 4 others (1985). Miscellaneous anticancer agents. In *Cancer Chemotherapy*, **7**, Pinedo, H.M. & Chabner, B.A. (eds), pp. 156–158. Elsevier: Amsterdam.

YOUNG, C.W., CURRIE, V.E., KIM, J.H., O'HEHIR, M.A., FARAG, F.M. & KINAHAN, J.E. (1981). Phase I and clinical pharmacologic evaluation of Lonidamine in patients with advanced cancer. *Oncology*, **41** (suppl 1), 60.

ZUPI, G., GRECO, C., LARDONIO, N., BENASSI, M., SILVESTRINI, B. & CAPUTO, A. (1986). *In vitro* and *in vivo* potentiation by Lonidamine of the antitumour effect of adriamycin. *Anticancer Res.*, **6**, 1245.